# Screening for Atrial Fibrillation – A Cross-Sectional Survey of Healthcare Professionals in Primary Care

**DOI:** 10.1371/journal.pone.0152086

**Published:** 2016-04-01

**Authors:** Jaspal S. Taggar, Tim Coleman, Sarah Lewis, Matthew Jones

**Affiliations:** 1 Division of Primary Care, University of Nottingham, Nottingham, United Kingdom; 2 Division of Epidemiology & Public Health, University of Nottingham, Nottingham, United Kingdom; University of Bologna, ITALY

## Abstract

**Introduction:**

Screening for atrial fibrillation (AF) in primary care has been recommended; however, the views of healthcare professionals (HCPs) are not known. This study aimed to determine the opinions of HCP about the feasibility of implementing screening within a primary care setting.

**Methods:**

A cross-sectional mixed methods census survey of 418 HCPs from 59 inner-city practices (Nottingham, UK) was conducted between October-December 2014. Postal and web-surveys ascertained data on existing methods, knowledge, skills, attitudes, barriers and facilitators to AF screening using Likert scale and open-ended questions. Responses, categorized according to HCP group, were summarized using proportions, adjusting for clustering by practice, with 95% C.Is and free-text responses using thematic analysis.

**Results:**

At least one General Practitioner (GP) responded from 48 (81%) practices. There were 212/418 (51%) respondents; 118/229 GPs, 67/129 nurses [50 practice nurses; 17 Nurse Practitioners (NPs)], 27/60 healthcare assistants (HCAs). 39/48 (81%) practices had an ECG machine and diagnosed AF in-house. Non-GP HCPs reported having less knowledge about ECG interpretation, diagnosing and treating AF than GPs. A greater proportion of non-GP HCPs reported they would benefit from ECG training specifically for AF diagnosis than GPs [proportion (95% CI) GPs: 11.9% (6.8–20.0); HCAs: 37.0% (21.7–55.5); nurses: 44.0% (30.0–59.0); NPs 41.2% (21.9–63.7)]. Barriers included time, workload and capacity to undertake screening activities, although training to diagnose and manage AF was a required facilitator.

**Conclusion:**

Inner-city general practices were found to have adequate access to resources for AF screening. There is enthusiasm by non-GP HCPs to up-skill in the diagnosis and management of AF and they may have a role in future AF screening. However, organisational barriers, such as lack of time, staff and capacity, should be overcome for AF screening to be feasibly implemented within primary care.

## Introduction

Screening for atrial fibrillation (AF), using opportunistic pulse palpation and confirmatory 12-lead electrocardiogram (ECG) in those with an irregular pulse, has been recommended as an intervention to improve the detection of this arrhythmia.[1–4] Based primarily on findings from the Screening for Atrial Fibrillation in the Elderly (SAFE) trial,[5, 6] this method of AF screening in primary care was found to be both effective and cost-effective for detecting incident AF in patients over 65 years of age. Combined with appropriate provision of anti-thrombotic therapy in patients with newly diagnosed AF, screening is likely to reduce the burden of thromboembolic stroke.[7]

Although AF screening has been recommended, the feasibility of introducing it into primary care is unclear and it is not known whether primary care has sufficient resources to deliver screening. Furthermore, understanding the opinions of healthcare professionals (HCPs) expected to undertake AF screening activities would be important to enable the identification of barriers, facilitators and needs for this key stakeholder group to deliver screening.

The gold standard test for identifying AF is a 12-lead ECG interpreted by a competent professional.[4] Consequently, the effectiveness of screening is dependent upon accurate ECG interpretation for diagnosing AF. A recent systematic review investigated the accuracy of different methods for interpreting 12-lead ECGs and diagnosing AF.[8] The authors found that, compared to cardiac specialist-diagnosed AF, automated software had the highest specificity but a low sensitivity, and was therefore likely to miss cases of AF. The sensitivities for primary care professionals were similar to automated software, although accuracy of diagnosing AF was lower for nurses than General Practitioners (GPs).[8] These findings suggest that 12-lead ECG interpretation by primary HCPs could be better and, if screening were implemented within primary care without addressing this issue, the effectiveness of screening could potentially be undermined. Consequently, training to improve the ability of HCPs in primary care to interpret ECGs and accurately diagnose AF would be an important consideration when designing and implementing any future AF screening programme.

To our knowledge there have been no studies that have investigated the feasibility of introducing AF screening into primary care and opinions of HCPs about conducting screening activities. Therefore, this study aimed to determine existing methods used for diagnosing AF within primary care, and to determine and compare the knowledge, skills and attitudes (KSA) and opinions of HCPs for AF screening within this setting.

## Materials and Methods

### Study Design and Study Participants

A cross-sectional census survey of HCPs in Nottingham City Clinical Commissioning Group (CCG) was conducted between October and December 2014. Nottingham City CCG comprised 67 inner-city GP practices serving 340,000 patients;[9] although the CCG has similar prevalence’s of long-term conditions to national estimates, there is greater mortality from cardio-respiratory diseases and greater potential years of life lost from causes amenable to healthcare than average estimates for England.[10] Prior to survey implementation, information from on-line public resources and Nottingham City CCG were used to create a list of HCPs working at each practice. Eligible participants were GPs, all nurses and healthcare assistants (HCAs). Practice managers were subsequently contacted by telephone to check record accuracy. Individual participants were provided study information and then surveyed using mixed methods of postal and web-survey. Implied consent was provided by participants by completion and submission of the survey; separate written or verbal consent was not obtained as implied consent was deemed appropriate and approved.

### Survey Design and Implementation

Participant characteristics were ascertained: the number of years practising as a HCP, whether participants worked full-time (number of days worked in those not working full-time), if ECG training had been received since graduation and the time since training in those previously receiving ECG training. The survey, consisting predominately of three and five-point Likert scale closed questions (S1 Survey), was used to ascertain information about existing methods for detecting and diagnosing AF, and participant knowledge, skills and attitudes (KSA) for these activities. The survey also included questions to ascertain participant views on enthusiasm and potential roles in future AF screening. Barriers and training related facilitators for AF screening were ascertained using open questions requiring free-text responses [*Are there any specific areas about the diagnosis of AF using 12-lead ECGs that you would like training*? *If such a screening program was introduced*, *what further training would you need to be able to undertake this role*? *If a screening program for AF was introduced*, *are there any problems you think might prevent it working effectively at your surgery*?*]*

The survey was initially piloted on HCPs from a different CCG than the intended population (five GPs, four Nurses and one HCA) and minor modifications were required.

Postal contact was made before survey implementation to inform participants about the research. A postal survey was then sent to all individuals; a web-link was also provided to enable on-line completion, if preferred. Two postal reminders (after four and eleven weeks) were sent to non-responders and, to promote greater awareness and improve response, the research team attended two CCG led practice learning time events during the survey period.

Approval of study materials and procedures was granted by the University of Nottingham Research & Ethics Committee (REF: B11092014 14085 SoM PC) and Nottingham City CCG Research and Development (REF: 159703).

### Statistical Analysis

Analyses were conducted using Stata version 11.0. Responses to survey questions, stratified by professional group, were summarised using proportions for categorical data and mean (SD) for parametric data, respectively.

GPs have a lead role in practice management and are likely to have the most accurate knowledge of existing methods for detecting AF; for questions that related to existing methods of diagnosing AF within the practice, analyses were conducted at a practice level and used only GP responses. Remaining questions were analysed within HCP occupation categories ascertained from the survey (GPs, nurses, nurse practitioners (NPs) and HCAs). NPs are registered nurses that work at a level well beyond initial registration with greater competencies and autonomy in patient care.[11]

Differences in participant characteristics across HCP groups were determined using chi-squared test, for categorical data, and analysis of variance (ANOVA) for parametric continuous data. Participant responses to questions about KSA to AF screening were summarized using proportions and 95% confidence intervals (CI) within HCP categories and allowed for the effects of clustering by practice using robust standard errors. Significance of associations between HCP groups was determined, when cell sizes were sufficient, using logistic or multinomial regression, for dichotomous or categorical variables respectively.

Open-ended questions were read independently by one researcher (JT) and a thematic analytical approach was used to determine major themes for barriers and facilitators for AF screening.[12]

## Results

### Response

**Fig 1:**
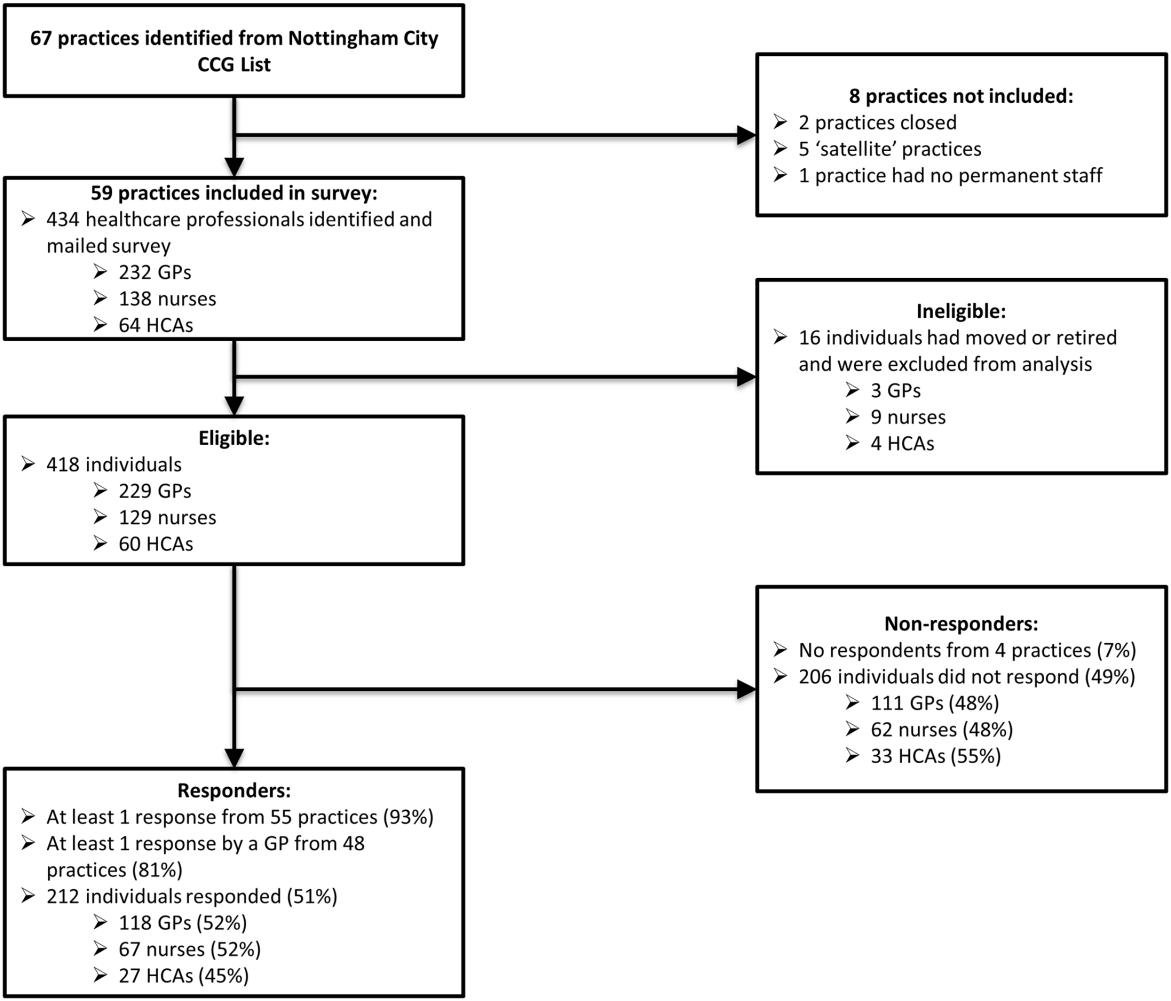
Participant response to the survey.

Participant response is shown in Fig 1. Of 67 practices registered within Nottingham City CCG, 59 were eligible for the survey; eight were excluded as they had closed, had no permanent staff, or shared staff with another practice. From 59 practices, there were 434 potentially eligible HCPs; 16 individuals were excluded because they were no longer employed by the CCG or had retired since initial contact was made. The final survey population was therefore 418 HCPs (229 GPs; 129 nurses; 60 HCAs). At least one GP responded from 48/59 (81%) practices; from all HCPs there were 212 (51%) respondents. [GPs: 52% (118/229); nurses: 52% (67/129); HCAs: 45% (27/60)]. Of the 67 nurse respondents, 17 were NPs. No duplicate surveys were returned.

### Participant Characteristics

GPs had worked for a mean (SD) 20.1 (9.2) years and the time in practice was similar for nurses and NPs. HCAs had worked for a significantly shorter time (mean (SD) of 11.2 (8.5) years; p<0.001). Full-time working was similar across categories of HCPs. However, of participants working part-time, there were significant differences in the number of days worked across HCP groups; mean (SD) days worked were 3.1 (0.7), 3.8 (1.1), 3.5 (1.1) and 3.7 (0.8) for GPs, HCAs, nurses and NPs (p = 0.009). Significantly more GPs (62.7%) and nurse practitioners (76.5%) received ECG training since graduation than nurses (50.0%) and HCAs (23.1%); p = 0.005. However, of those receiving ECG training since graduation, a greater proportion of GPs (66.2%), HCAs (83.3%) and NPs (69.2%) received it within the last five years compared to nurses (28.0%); p = 0.014).

### Existing Methods for Diagnosing AF

From 48 practices with at least one GP respondent, 39 (81%) reported having an ECG machine. In practices without an ECG machine, all (100%) reported using another NHS GP practice to obtain ECGs and a few (12.5%) also used NHS hospitals. In practices with an ECG machine, HCAs and nurses (89.7% and 82.1% of practices, respectively) were most often reported as the HCPs responsible for conducting ECGs. GPs conducted ECGs in only 12.8% practices. 81.3% of practices reported diagnosing AF in-house and, in all those practices, GPs were responsible for making AF diagnoses. NPs were also reported to diagnose AF in 15.4% practices. Only 37.5% practices reported always diagnosing AF in-house. In practices that did not always make AF diagnoses most used other NHS services for this; 60% reported using an NHS hospital and 6.7% used other GP practices. 6.7% practices reported using private healthcare providers to diagnose AF and the remainder did not know or respond.

### Knowledge, Skills and Attitudes (KSA) Relating to AF Screening

Table 1 presents the results for the knowledge and skills of HCPs for AF screening. There were no substantial differences between HCPs for performing pulse checks routinely this activity was conducted by 95.8% GPs, 88.9% HCAs, 94.0% nurses and 100% NPs. There were no substantial differences in how often pulse checks were performed by HCPs although a greater proportion of NPs reported always undertaking this activity. However, fewer HCAs (33.3% (95% CI 18.2–52.9)) were confident at performing pulse checks than other HCP groups. A greater proportion of non-GP HCPs were confident at performing 12-lead ECGs than GPs [Proportion (95% CI) for HCAs: 77.8% (56.7–90.4); nurses: 70.0% (54.4–82.0); NPs: 94.1% (66.0–99.2); GPs: 33.1% (23.7–44.0)]. Fewer nurses and HCAs were confident at diagnosing AF using 12-lead ECG than GPs and NPs.

**Table 1:** Knowledge and skills in conducting AF screening actives by healthcare professionals.

Question	Response	GP (N = 118)		Healthcare assistant (N = 27)		Nurse (N = 50)		Nurse practitioner (N = 17)		P-value
		N (%)*	95% C.I*	N (%)*	95% C.I*	N (%)*	95% C.I*	N (%)*	95% C.I*	
**Pulse checks**										
Perform pulse checks	Yes	113 (95.8)	89.9–98.3	24 (88.9)	69.3–96.7	47 (94.0)	82.3–98.1	17 (100.0)	-	0.210
	No	4 (3.4)	1.2–8.9	3 (11.1)	3.4–30.7	3 (6.0)	1.9–17.0	0 (0.0)	-	
How often pulse check performed	Always	24 (20.3)	13.4–29.6	10 (37.0)	20.4–57.5	13 (26.0)	15.9–39.4	10 (58.8)	35.2–78.9	<0.001
	Often	64 (54.2)	45.5–62.7	7 (25.9)	11.2–49.2	24 (48.0)	35.5–60.8	6 (35.3)	19.3–55.5	
	Sometimes	23 (19.5)	13.2–27.8	7 (25.9)	12.0–47.2	10 (20.0)	11.8–31.9	1 (5.9)	0.9–31.9	
	Rarely	1 (0.8)	0.1–6.2	0 (0.0)	-	0 (0.0)	-	0 (0.0)	-	
**Confidence in performing screening activities**										
Identifying an irregular pulse	Very confident	99 (83.9)	77.0–89.0	9 (33.3)	18.2–52.9	36 (72.0)	55.6–84.1	17 (100.0)	-	n/a
	Somewhat confident	18 (15.3)	10.1–22.2	14 (51.9)	32.5–70.7	12 (24.0)	12.7–40.6	0 (0.0)	-	
	Not confident at all	0 (0.0)	-	3 (11.1)	3.8–28.5	1 (2.0)	0.3–13.9	0 (0.0)	-	
Performing 12-lead ECG	Very confident	39 (33.1)	23.7–44.0	20 (77.8)	56.7–90.4	35 (70.0)	54.4–82.0	16 (94.1)	66.0–99.2	<0.001
	Somewhat confident	53 (44.9)	35.3–54.9	3 (11.1)	3.4–30.7	10 (20.0)	10.8–34.0	1 (5.9)	0.8–34.0	
	Not confident at all	25 (21.2)	15.4–28.5	2 (7.4)	1.7–26.6	4 (8.0)	2.9–20.0	0 (0.0)	-	
Deciding if ECG shows AF	Very confident	65 (55.1)	46.1–63.8	0 (0.0)	-	5 (10.0)	4.5–20.8	5 (29.4)	10.5–59.6	<0.001
	Somewhat confident	50 (42.4)	33.6–51.6	5 (18.5)	8.4–35.9	19 (38.0)	25.9–51.8	5 (29.4)	12.5–54.8	
	Not confident at all	1 (0.8)	0.1–6.1	19 (74.1)	56.0–86.5	25 (50.0)	36.3–63.7	7 (41.2)	19.5–66.9	
**Knowledge of performing screening activities**										
Identifying an irregular pulse	Excellent	57 (48.3)	38.7–58.1	8 (29.6)	14.7–50.6	23 (46.0)	32.4–60.2	13 (76.5)	46.5–92.4	n/a
	Good	58 (49.2)	39.4–59.0	11 (40.7)	21.7–63.0	24 (48.0)	35.5–60.8	4 (23.5)	7.6–53.5	
	Fair	2 (1.7)	0.4–6.7	5 (22.2)	9.5–43.9	2 (4.0)	1.0–14.5	0 (0.0)	-	
	Poor	0 (0.0)	-	1 (3.7)	0.5–23.5	0 (0.0)	-	0 (0.0)	-	
	Non-existent	0 (0.0)	-	0 (0.0)	-	0 (0.0)	-	0 (0.0)	-	
Deciding the cause of an abnormal 12-lead ECG	Excellent	7 (5.9)	2.8–12.0	0 (0.0)	-	1 (2.0)	0.3–13.9	2 (11.8)	3.0–36.4	<0.001
	Good	56 (47.5)	38.8–56.3	1 (3.7)	0.6–20.7	6 (12.0)	4.9–26.4	3 (17.6)	5.8–42.6	
	Fair	48 (40.7)	31.2–50.9	5 (18.5)	6.8–41.6	17 (34.0)	22.9–47.2	5 (29.4)	13.6–52.4	
	Poor	5 (4.2)	1.5–11.7	7 (25.9)	13.5–44.0	17 (34.0)	21.4–49.3	7 (41.2)	21.9–63.7	
	Non-existent	0 (0.0)	-	12 (44.4)	26.5–63.9	8 (16.0)	6.6–34.0	0 (0.0)	-	
Deciding if 12-lead ECG shows AF	Excellent	31 (26.3)	17.8–37.0	0 (0.0)	-	1 (2.0)	0.3–12.9	2 (11.8)	2.7–38.8	n/a
	Good	74 (62.7)	51.5–72.7	3 (11.1)	2.5–37.8	8 (16.0)	8.3–28.7	6 (35.3)	15.3–62.3	
	Fair	11 (9.3)	4.7–17.8	7 (25.9)	13.5–44.0	20 (40.0)	29.6–51.4	4 (23.5)	9.9–46.4	
	Poor	0 (0.0)	-	5 (18.5)	7.3–39.6	15 (30.0)	18.4–44.8	4 (23.5)	9.0–48.8	
	Non-existent	0 (0.0)	-	9 (33.3)	16.9–55.1	5 (10.0)	4.1–22.3	1 (5.9)	0.8–34.0	
Deciding on treatment for AF	Excellent	20 (16.9)	9.9–27.4	0 (0.0)	-	0 (0.0)	-	1 (5.9)	0.8–34.0	<0.001
	Good	66 (55.9)	46.3–65.2	1 (3.7)	0.5–23.5	8 (16.0)	7.7–30.3	4 (23.5)	7.1–55.4	
	Fair	27 (22.9)	16.1–31.5	1 (3.7)	0.5–22.1	12 (24.0)	13.8–38.4	4 (23.5)	9.0–48.8	
	Poor	2 (1.7)	0.4–7.0	3 (14.8)	5.6–34.0	16 (32.0)	20.0–47.0	6 (35.3)	15.3–62.3	
	Non-existent	1 (0.8)	0.1–6.1	19 (70.4)	47.3–86.3	12 (24.0)	12.9–40.2	2 (11.8)	2.7–38.8	

*N = number of participants responding to question item; % = proportion of participants, adjusted for clustering by practice; 95% C.I = 95% confidence interval for the proportion of participants; n/a = unable to calculate p-value to insufficient data within cells

Missing data within question responses is present when the sum of column percentages <100%

Only 29.6% (95% CI 14.7–50.6) HCAs reported having excellent knowledge about identifying an irregular pulse, which was lower than other HCP groups [proportion (95% CI) for GPs 48.3 (38.7–58.1); nurses: 46.0 (32.4–60.2); NPs 76.5 (46.5–92.4)]. Fewer non-GP HCPs reported having excellent or good knowledge for interpreting abnormal 12-lead ECGs, diagnosing and treating AF than GPs.

Attitudes of HCPs about training for AF screening are presented in Table 2. More HCAs (48.1% (95% CI 30.6–66.2)) felt they would benefit from pulse palpation training than other HCPs (proportion (95% CI) for GPs: 7.6% (3.6–15.3); nurses: 18.0% (8.5–34.0); NPs: 0%]. HCPs felt they would benefit from ECG interpretation training and there were no substantial differences between professional groups. However, a greater proportion of non-GP HCPs reported they would benefit from ECG interpretation training specifically for AF than GPs [proportion (95% CI) for GPs: 11.9% (6.8–20.0); HCAs: 37.0% (21.7–55.5); nurses: 44.0% (30.0–59.0); NPs 41.2% (21.9–63.7)]. More non-GP HCPs also felt they would be better at diagnosing AF if they received ECG interpretation training than GPs. Similar proportions of HCPs reported enthusiasm to receive general ECG training across professional groups. However, more non-GP HCPs were enthusiastic to receive ECG training specifically for AF than GPs [proportion (95% CI) for GPs: 13.6 (8.2–21.5); HCAs: 40.7% (24.2–59.7); nurses 38.0% (24.1–54.1); NPs: 29.4% (13.6–52.4); p<0.001). In contrast, fewer HCAs, nurses and NPs wanted to be involved in diagnosing AF than GPs.

**Table 2:** Attitudes of healthcare professionals about training for AF screening.

Question	Response	GP (N = 118)		Healthcare assistant (N = 27)		Nurse (N = 50)		Nurse practitioner (N = 17)		P-value
		N (%)*	95% C.I*	N (%)*	95% C.I*	N (%)*	95% C.I*	N (%)*	95% C.I*	
Benefit from pulse palpation training	Strongly agree	9 (7.6)	3.6–15.3	13 (48.1)	30.6–66.2	9 (18.0)	8.5–34.0	0 (0.0)	-	n/a
	Agree	16 (13.6)	8.2–21.6	9 (33.3)	18.2–52.9	17 (34.0)	22.3–48.1	1 (5.9)	0.8–34.0	
	Not sure	16 (13.6)	8.5–21.0	0 (0.0)	-	5 (10.0)	4.3–21.5	3 (17.6)	5.8–42.6	
	Disagree	56 (47.5)	38.0–57.1	1 (3.7)	0.5–23.5	14 (28.0)	16.8–42.9	8 (47.1)	25.9–69.3	
	Strongly disagree	19 (16.1)	10.5–23.9	1 (3.7)	0.6–20.7	4 (8.0)	3.1–18.9	5 (29.4)	12.5–54.8	
Benefit from ECG interpretation training	Strongly agree	37 (31.4)	23.1–50.0	15 (55.6)	37.0–72.7	25 (50.0)	35.8–64.2	10 (58.8)	36.3–78.1	n/a
	Agree	60 (50.8)	41.3–60.4	6 (22.2)	10.9–40.0	17 (34.0)	21.6–49.0	5 (29.4)	13.6–52.4	
	Not sure	7 (5.9)	3.3–10.6	2 (7.4)	1.0–38.9	3 (6.0)	2.0–16.7	0 (0.0)	-	
	Disagree	8 (6.8)	3.1–14.3	2 (7.4)	1.7–26.6	2 (4.0)	1.0–14.9	2 (11.8)	2.7–38.8	
	Strongly disagree	4 (3.4)	1.3–8.4	0 (0.0)	-	2 (4.0)	1.0–15.2	0 (0.0)	-	
Benefit for ECG interpretation training for AF	Strongly agree	14 (11.9)	6.8–20.0	10 (37.0)	21.7–55.5	22 (44.0)	30.0–59.0	7 (41.2)	21.9–63.7	<0.001
	Agree	38 (32.2)	25.3–40.0	9 (33.3)	18.2–52.9	17 (34.0)	21.3–49.4	7 (41.2)	21.1–64.7	
	Not sure	15 (12.7)	8.1–19.3	5 (18.5)	6.5–42.6	3 (6.0)	2.0–16.7	2 (11.8)	2.7–38.8	
	Disagree	42 (35.6)	27.2–44.9	1 (3.7)	0.5–23.5	5 (10.0)	4.3–21.5	1 (5.9)	0.83–4.0	
	Strongly disagree	7 (5.9)	3.0–11.4	0 (0.0)	-	2 (4.0)	1.015.2	0 (0.0)	-	
Better at diagnosing AF if received ECG interpretation training	Strongly agree	20 (16.9)	10.7–25.7	11 (40.7)	24.6–59.1	19 (38.0)	24.5–53.7	7 (41.2)	21.9–63.7	<0.001
	Agree	31 (26.3)	19.6–34.2	5 (18.5)	7.7–38.2	20 (40.0)	27.2–54.4	8 (47.1)	25.9–69.3	
	Not sure	22 (18.6)	11.3–29.2	5 (18.5)	7.0–40.7	4 (8.0)	2.9–20.4	1 (5.9)	0.8–34.0	
	Disagree	35 (29.7)	21.1–39.9	2 (7.4)	1.7–26.6	3 (6.0)	1.9–17.0	1 (5.9)	0.8–34.0	
	Strongly disagree	8 (6.8)	3.5–12.7	1 (3.7)	0.5–23.5	3 (6.0)	1.9–17.0	0 (0.0)	-	
Would like ECG training (any condition)	Strongly agree	37 (31.4)	23.9–39.9	12 (44.4)	27.3–63.0	21 (42.0)	28.1–57.3	11 (64.7)	44.5–80.7	n/a
	Agree	61 (51.7)	42.5–60.8	6 (22.2)	10.9–40.0	16 (32.0)	19.2–48.2	5 (29.4)	14.9–49.8	
	Not sure	8 (6.8)	3.5–12.8	2 (7.4)	1.7–26.6	5 (10.0)	3.7–24.0	0 (0.0)	-	
	Disagree	7 (5.9)	2.9–11.8	3 (11.1)	2.5–37.8	3 (6.0)	1.9–17.4	1 (5.9)	0.8–34.0	
	Strongly disagree	3 (2.5)	0.9–7.3	0 (0.0)	-	4 (8.0)	3.1–19.3	0 (0.0)	-	
Would like ECG training (AF)	Strongly agree	16 (13.6)	8.2–21.5	11 (40.7)	24.2–59.7	19 (38.0)	24.1–54.1	5 (29.4)	13.6–52.4	<0.001
	Agree	33 (28.0)	21.4–35.6	7 (25.9)	13.5–44.0	14 (28.0)	17.3–42.0	9 (52.9)	31.0–73.8	
	Not sure	21 (17.8)	11.5–26.5	3 (11.1)	3.6–29.7	5 (10.0)	4.6–20.4	2 (11.8)	2.7–38.8	
	Disagree	38 (32.2)	23.1–42.8	2 (7.4)	1.8–25.4	8 (16.0)	8.3–28.7	1 (5.9)	0.8–34.0	
	Strongly disagree	8 (6.8)	3.4–13.2	0 (0.0)	-	3 (6.0)	1.9–17.0	0 (0.0)	-	
Would like to be involved in diagnosing AF	Strongly agree	32 (27.1)	19.5–36.4	6 (22.2)	10.0–42.3	12 (24.0)	14.2–37.6	8 (47.1)	26.2–69.0	<0.001
	Agree	61 (51.7)	43.8–59.5	3 (11.1)	3.6–29.7	10 (20.0)	11.0–33.6	2 (11.8)	3.0–36.4	
	Not sure	8 (6.8)	3.0–14.5	6 (22.2)	9.8–42.8	14 (28.0)	16.5–43.3	6 (35.3)	17.6–58.1	
	Disagree	9 (7.6)	3.9–14.4	4 (14.8)	5.6–34.0	10 (20.0)	10.3–35.1	1 (5.9)	0.9–31.1	
	Strongly disagree	2 (1.7)	0.4–6.9	4 (14.8)	5.8–32.9	3 (6.0)	1.9–17.0	0 (0.0)	-	

*N = number of participants responding to question item; % = proportion of participants, adjusted for clustering by practice; 95% C.I = 95% confidence interval for the proportion of participants; n/a = unable to calculate p-value to insufficient data within cells

Missing data within question responses is present when the sum of column percentages <100%

### Facilitators and Barriers to AF Screening

HCPs views on their potential roles in AF screening are presented in Table 3. Most participants reported having a likely role in performing pulse checks although a greater proportion of nurses and NPs reported having this role than other HCPs. More nurses and NPs also reported being very likely to have a role in conducting 12-lead ECGs [proportion (95% CI) for GPs: 31.4% (23.1–41.0); HCAs: 48.1% (30.4–66.4); nurses: 70.0% (52.7–83.0); NPs 64.7% (39.9–83.5)]. Fewer non-GP HCPs reported having a future role in ECG interpretation and AF diagnosis than GPs.

**Table 3:** Perceived role of healthcare professionals in future AF screening.

Question	Response	GP (N = 118)		Healthcare assistant (N = 27)		Nurse (N = 50)		Nurse practitioner (N = 17)		P-value
		N (%)*	95% C.I*	N (%)*	95% C.I*	N (%)*	95% C.I*	N (%)*	95% C.I*	
Role in performing pulse checks	Very likely	61 (51.7)	41.0–62.2	12 (44.4)	27.6–62.7	42 (84.0)	72.5–91.3	12 (70.6)	44.4–87.8	n/a
	Likely	37 (31.4)	22.9–41.3	7 (25.9)	11.2–49.2	6 (12.0)	5.8–23.3	2 (11.8)	1.6–52.3	
	Unsure	9 (7.6)	3.9–14.4	6 (22.2)	9.8–42.8	0 (0.0)	-	0 (0.0)	-	
	Unlikely	3 (2.5)	0.8–7.5	0 (0.0)	-	0 (0.0)	-	1 (5.9)	0.8–34.0	
	Very unlikely	3 (2.5)	0.8–22.2	0 (0.0)	-	0 (0.0)	-	0 (0.0)	-	
Role in conducting 12-lead ECGs	Very likely	37 (31.4)	23.1–41.0	13 (48.1)	30.4–66.4	35 (70.0)	52.7–83.0	11 (64.7)	39.9–83.5	n/a
	Likely	29 (24.6)	17.1–33.9	6 (22.2)	9.6–43.3	4 (8.0)	2.9–20.0	3 (17.6)	4.0–52.2	
	Unsure	6 (5.1)	2.3–11.0	4 (14.8)	6.1–31.7	4 (8.0)	3.0–19.3	0 (0)	-	
	Unlikely	27 (22.9)	16.4–31.0	0 (0)	-	1 (2.0)	0.3–13.4	1 (5.9)	0.8–34.0	
	Very unlikely	14 (11.9)	7.7–17.7	2 (7.4)	1.7–26.6	4 (8.0)	2.9–20.0	0 (0)	-	
Role in ECG interpretation for AF	Very likely	71 (60.2)	48.8–70.5	2 (7.4)	1.7–26.6	7 (14.0)	6.4–27.8	5 (29.4)	12.5–54.8	<0.001
	Likely	37 (31.4)	22.8–41.5	2 (7.4)	1.7–26.6	8 (16.0)	8.1–29.1	4 (23.5)	8.3–51.0	
	Unsure	6 (5.1)	2.3–10.8	9 (33.3)	18.9–51.7	18 (36.0)	24.4–49.5	3 (17.6)	5.8–42.6	
	Unlikely	0 (0)	-	6 (22.2)	9.8–42.8	9 (18.0)	10.5–29.0	3 (17.6)	4.0–52.2	
	Very unlikely	0 (0)	-	6 (22.2)	10.4–41.1	6 (12.0)	5.4–24.5	0 (0)	-	
Role in diagnosing AF	Very likely	69 (58.5)	46.9–69.2	0 (0)	-	5 (10.0)	3.7–24.1	5 (29.4)	12.5–54.8	n/a
	Likely	40 (33.9)	24.8–44.4	1 (3.7)	0.5–23.5	6 (12.0)	5.2–25.2	3 (17.6)	5.3–44.9	
	Unsure	5 (4.2)	1.7–9.9	8 (29.6)	16.2–47.9	15 (30.0)	19.3–43.4	1 (5.9)	0.8–34.0	
	Unlikely	0 (0)	-	3 (11.1)	2.5–37.8	11 (22.0)	11.8–37.2	6 (35.3)	15.3–62.3	
	Very unlikely	0 (0)	-	13 (48.1)	30.5–66.3	11 (22.0)	12.0–36.8	0 (0)	-	

*N = number of participants responding to question item; % = proportion of participants, adjusted for clustering by practice; 95% C.I = 95% confidence interval for the proportion of participants; n/a = unable to calculate p-value to insufficient data within cells

Missing data within question responses is present when the sum of column percentages <100%

There were 337 free-text responses from 171/212 (81%) respondents (105 GPs; 13 HCAs; 53 nurses). Around 20% responses identified no barriers to screening within current practice. Common themes for barriers, in all HCP groups, to AF screening were time to undertake screening, workload, lack of appointments, staffing levels within the practice, access to the required equipment, and available funding to conduct screening activities. [Comment 212 (GP): “we would require some form of extra resources to carry this out depending on the work required general practice is currently overstretched with work and conflicting demands”; Comment 219 (GP) “workload issues”; Comment 231 (GP): time, time, time, the waiting time for anticoagulation clinic would need to be reduced currently two to three weeks and GP carries responsibility for any adverse event; also who will find the money for new anticoagulants”. Comment 311 (Nurse): “lack of appointments, too few nurses, extra load on all members of the team”]. Less common barriers included the perception that screening activities were not their current role, lack of space within the practice, lack of training, and the patient reluctance to screening.

Only 10% of responses suggested there were no facilitators required for screening to be implemented within existing practice. The most common theme identified as a facilitator for screening was additional training requirements; commonly reported requirements were training for conducting and interpreting 12-lead ECGs, the management of AF and undertaking pulse palpation. [Comment 57 (GP): “brief ECG update training and advice on management of AF once diagnosed”; Comment 69 (GP): Training for practice nurses in AF diagnosis/management; written protocol pathway to aid above process”; Comment 99 (GP): Further training on ECG interpretation. I am fairly confident that I can identify AF on an ECG but looking at ECG uncovers other abnormalities that I have less confidence in my interpretation”; Comment 151 (Nurse): ECG training reading and interpretation of results”]. Less common facilitators to screening included provision or access to 12-lead ECGs and guidelines on AF screening.

## Discussion

We found that, even in this inner-city area, most practices are able to perform and interpret ECGs in-house and were potentially well-equipped for future AF screening. Non-GP HPs reported having less knowledge about ECG interpretation and the treatment of AF than GPs. However, non-GP HCPs more frequently reported they would benefit from ECG training specifically for diagnosing AF. There was enthusiasm for training for ECG interpretation by all HCPs but was specifically for AF diagnosis in non-GP groups. However, non-GP HCPs did not perceive themselves to have a future role in ECG interpretation or AF diagnosis.

### Strengths and Limitations

To our knowledge, this is the first study to ascertain readiness for and views of HCPs regarding the introduction of AF screening in primary care. A strength of this study was the high practice-level response rate: at least one GP responded from 81% of practices; therefore we are likely to have ascertained representative estimates for existing methods for detecting AF within inner-city practices. Whilst the response rate from individual participants was satisfactory (51%) there is a possibility that non-respondents’ knowledge, skills, attitudes and opinions might be different from those who completed questionnaires. For example, non-responders may have lower enthusiasm for AF screening and we may have overestimated HCP interest in this.

Although findings are likely to represent the views of HCPs within inner-city practices of Nottingham City CCG, another limitation of this study is the generalizability of findings to professionals in other primary care settings, such as those working in rural settings. The prevalence and daily health life problems experienced by patients from long-term conditions in Nottingham CCG is similar to national average estimates.[13] Moreover, in 2014 Nottingham City CCG had similar ratios of GPs and nurses to patient population as the England average.[14] This suggests the burden of long-term conditions and staffing available for managing these is similar to national estimates. Consequently, the views of HCPs in our study may be generalizable to professionals from other inner-city practices.

HCPs working in the same practice could have similar opinions but we allowed for this by adjusting for clustering by practice and, consequently, the outcomes from our study are likely to have greater precision. Although p-values were obtained to give an indication of true differences between HCP groups, the level of significance should be treated with caution given the number of statistical tests performed.

### Comparison with Existing Literature

Although not implemented, AF screening has been recommended for identifying new cases of AF. [15],[16] Our survey suggests primary care is potentially well-equipped and ready for delivering AF screening with good access to conducting and interpreting ECGs. Moreover, all HCPs felt they were able to perform pulse palpation. It may therefore be feasible to deliver AF screening within this setting.

Screening for AF in primary care would result in a substantial increase in the number of ECGs conducted and that require interpretation. Our findings suggest that non-GP HCPs could have an important role in this. Although non-GP HCPs reported deficiencies in knowledge for ECG interpretation than GPs, we found they had enthusiasm to undertake ECG training specifically for AF diagnosis. Furthermore, we identified training to interpret ECGs and manage AF as a facilitator for screening although across HCP groups. Nurses may have the greatest potential for supporting AF screening. Nurses are having a greater role in managing long-term conditions, and research suggests that nurses prefer increased healthcare responsibilities, having an important role in disease management.[17],[18] Studies have also found that, with appropriate training, the accuracy of ECG interpretation by nurses can be improved.[19, 20]

Paradoxically, in our survey nurses reported the lack of a perceived future role in AF diagnosis and management despite reporting enthusiasm for ECG interpretation training. This may be due to nurses sometimes seeing their role in clinical practice as vague[21]. We also identified a number of barriers to AF screening, particularly relating to lack of workforce and capacity to undertake screening, which may influence nurses’ lack of perceived role in future service delivery.

The barriers to AF screening that our study identified included lack of capacity, time, staff and funding to undertake screening activities within practices. Similar themes have been identified in studies investigating the introduction of screening for other conditions within primary care.[22–24] Furthermore, primary care in the UK is currently perceived to be in crisis, with surgeries facing cuts in funding,[25–27] poor recruitment[28], and reduced job satisfaction reported by GPs.[27, 29] Any future AF screening programme would have financial and staffing implications to GP surgeries and overcoming these barriers, in addition to facilitation of ECG interpretation training, would be imperative to ensure the successful implementation of this intervention.

## Conclusions

Primary care is potentially well-equipped and ready to deliver AF screening, with most practices having the ability to detect pulse irregularities, perform and interpret 12-lead ECGs. Compared to GPs, other HCPs report less knowledge and skills for interpreting 12-lead ECGs and diagnosing AF. However, there is enthusiasm by non-GP HCPs to gain skills in ECG interpretation. Nurses may have the greatest potential to up-skill and could have an important role in supporting future AF screening. However, many barriers to AF screening exist including lack of practice capacity, staffing and funding, and overcoming such barriers would be imperative to enable screening implementation.

## Supporting Information

S1 Data Dataset for analyses. (XLSX)

S1 Survey Survey for healthcare professionals in primary care about screening for atrial fibrillation. (DOCX)
