# Supplementary material for: Screening for Atrial Fibrillation – A Cross-Sectional Survey of Healthcare Professionals in Primary Care
Source: PLoS One. 2016 Apr 1;11(4):e0152086. doi: 10.1371/journal.pone.0152086 (PMC4818037; doi:10.1371/journal.pone.0152086)
Supplement: S1 Survey — (DOCX) [file pone.0152086.s002.docx]

**Your views on detecting and diagnosing atrial fibrillation (AF) in primary care**

*Please complete the following questionnaire by ticking the boxes that are most appropriate to you or by writing in the spaces provided. All information given is confidential. Unless stated, please only tick one box per question.*

**SECTION 1: About you**

1. Are you a:

General practitioner

Nurse practitioner

Nurse

Healthcare assistant

1. How many years have you been practising as a healthcare professional (**nearest whole years**)?
2. Do you currently work full time in general practice?

Yes **SKIP TO QUESTION 5** No

a

1. If part time, how many **days** do you work in a normal week?

**PLEASE TURN OVER**

**SECTION 2: About your current practice**

1. Do you perform pulse checks to identify if a patient has an irregular pulse?

Yes No **SKIP TO QUESTION 7**

a

1. How often do you perform pulse checks to identify an irregular pulse?

a

Always

Often

a

Sometimes

a

Rarely

a

1. Does your GP surgery have a 12-lead ECG machine?

Yes No **SKIP TO QUESTION 15**

1. Who carries out the 12-lead ECG on patients to obtain a heart tracing at your practice (*tick all those which are applicable*)?

General practitioner

Nurse practitioner

Nurse

Healthcare assistant

Other (**please specify**):

1. Is the decision about whether a 12-lead ECG shows AF made within your practice?

Yes No **SKIP TO QUESTION 14**

1. If yes, how often is this decision made at your practice:

a

Always **SKIP TO QUESTION 13**

Often

a

Sometimes

a

Rarely

a

Don’t know

a

**PLEASE TURN OVER**

1. Where else is the decision about whether a 12-lead ECG shows AF made (*tick all those which are applicable*)?

Another NHS GP practice

NHS hospital

Private health care provider

Other (**please specify**):

Don’t know

1. Which health professional at your practice usually makes the decision about whether a 12-lead ECG shows AF (*tick all those which are applicable*)?

General practitioner

Nurse practitioner

Nurse

Healthcare assistant

Other (**please specify**):

Don’t know

**PLEASE SKIP TO QUESTION 21**

1. If no, where is the decision about whether a 12-lead ECG shows AF made (*tick all those which are applicable*)?

Another NHS GP practice

NHS hospital

Private health care provider

Other (**please specify**):

Don’t know

**PLEASE SKIP TO QUESTION 21**

**PLEASE TURN OVER**

1. If you DON’T have a 12-lead ECG machine at your practice, how do you obtain a 12-lead ECG on your patients (*tick all those which are applicable*)?

Another NHS GP practice

NHS hospital

Private health care provider

Other (**please specify**):

Don’t know

1. Is the decision about whether a 12-lead ECG shows AF made within your practice?

Yes No **SKIP TO QUESTION 20**

a

1. If yes, how often is the decision about whether a 12-lead ECG shows AF made at your practice:

Always **SKIP TO QUESTION 19**

a

Often

a

Sometimes

a

Rarely

a

Don’t know

a

1. Where else is the decision about whether a 12-lead ECG shows AF made (*tick all those which are applicable*)?

Another NHS GP practice

NHS hospital

Private health care provider

Other (**please specify**):

Don’t know

1. At your practice, which health professional usually makes the decision about whether a 12-lead ECG shows AF (*tick all those which are applicable)*?

General practitioner

Nurse practitioner

Nurse

Healthcare assistant

Other (please specify):

Don’t know

**PLEASE SKIP TO QUESTION 21**

1. If no, where is the decision about whether an 12-lead ECG shows AF made (*tick all those which are applicable*)?

Another NHS GP practice

NHS hospital

Private health care provider

Other (**please specify**):

Don’t know

**PLEASE TURN OVER**

**SECTION 3: Your thoughts on training for diagnosing AF**

1. Since you have graduated/become a healthcare professional have you received any additional training about how to interpret 12-lead ECGs?

Yes No **SKIP TO QUESTION 23**

a

1. How long ago did you receive this?

Less than one year ago

a

Between one and five years ago

a

More than five years ago

a

1. How confident do you feel in performing the following tasks? (*Tick* ***one*** *box only for each question)*

|  | Very confident | Somewhat confident | Not confident at all |
| --- | --- | --- | --- |
| Identifying an irregular pulse using pulse palpation |  |  |  |
| Performing a 12-lead ECG |  |  |  |
| Deciding if a 12-lead ECG shows AF |  |  |  |

**PLEASE TURN OVER**

1. How would you rate your knowledge about the following tasks? (*Tick* ***one*** *box only for each question*)

|  | Excellent | Good | Fair | Poor | Non-existent |
| --- | --- | --- | --- | --- | --- |
| Identifying patients with an irregular pulse |  |  |  |  |  |
| Deciding about the cause of an abnormal 12-lead ECG |  |  |  |  |  |
| Deciding if a 12-lead ECG shows AF |  |  |  |  |  |
| Deciding on the treatment of AF once it has been diagnosed |  |  |  |  |  |

**PLEASE TURN OVER**

1. We’re interested in how you feel about atrial fibrillation. Below are a series of statements on this subject.

*Please tick* ***one*** *box for your most appropriate response to each statement*

|  | Strongly agree | Agree | Not sure | Disagree | Strongly disagree |
| --- | --- | --- | --- | --- | --- |
| I would benefit from further training on how to identify an irregular pulse in patients |  |  |  |  |  |
| I would benefit from further training about how to interpret a 12-lead ECG for any condition |  |  |  |  |  |
| I would benefit from further training about how to decide if a 12-lead ECG shows AF |  |  |  |  |  |
| I would be better at diagnosing AF if I received training about how to interpret a 12-lead ECG |  |  |  |  |  |
| I would like to receive training on how to interpret a 12-lead ECG for any condition |  |  |  |  |  |
| I would like to receive training on how to decide if a 12-lead ECG shows AF |  |  |  |  |  |
| I would like to be involved in diagnosing patients with AF |  |  |  |  |  |

1. Are there any specific areas about the diagnosis of AF using 12-lead ECGs that you would like training?

**PLEASE TURN OVER**

**SECTION 4: Your thoughts on screening for AF**

*It has been suggested that screening for AF in primary care is a relatively effective and cost-effective way of identifying people with AF. Current guidelines suggest that screening should involve using pulse palpation to identify patients with an irregular pulse, and then a 12-lead ECG should be conducted to determine whether the irregular pulse is caused by AF.*

1. If your practice was compelled to take part in such a national screening program, what role could you see yourself having in this?

|  | Very likely | Likely | Unsure | Unlikely | Very unlikely |
| --- | --- | --- | --- | --- | --- |
| Conduct pulse checks to identify an irregular pulse |  |  |  |  |  |
| Conduct 12-lead ECGs on patients with an irregular pulse |  |  |  |  |  |
| Deciding if a 12-lead ECG shows AF |  |  |  |  |  |
| Making the diagnosis of AF in a patient |  |  |  |  |  |

1. If such a screening program was introduced, what further training would you need to be able to undertake this role?

1. If a screening program for AF was introduced, are there any problems you think might prevent it working effectively at your surgery?

**PLEASE TURN OVER**

1. Would you like to receive a summary of the survey results?

Yes No

1. Would you like to be contacted about participating in future research about AF?

Yes No

1. If yes, please leave your contact details below:

**THANK YOU FOR COMPLETING THIS QUESTIONNAIRE**
